# Supplementary material for: Mitochondrial Calcium-Triggered Oxidative Stress and Developmental Defects in Dopaminergic Neurons Differentiated from Deciduous Teeth-Derived Dental Pulp Stem Cells with MFF Insufficiency
Source: Antioxidants (Basel). 2022 Jul 13;11(7):1361. doi: 10.3390/antiox11071361 (PMC9311869; doi:10.3390/antiox11071361)
Supplement: Supplementary file 1 [file antioxidants-11-01361-s001.zip › antioxidants-1771783-supple.pdf]

Table S1. Alexa Fluor-conjugated secondary antibodies.

Alexa Fluor-conjugated secondary antibodies	Catalog numbers
Alexa Fluor™ 488 Goat anti-Mouse IgG2a	#A21131
Alexa Fluor™ 488 Goat anti-Mouse IgG2b	#A21141
Alexa Fluor™ 488 Goat anti-Mouse IgG(H+L)	#A11001
Alexa Fluor™ 594 Goat anti-Mouse IgG1	#A21125
Alexa Fluor™ 594 Goat anti-Rabbit IgG(H+L)	#A27016

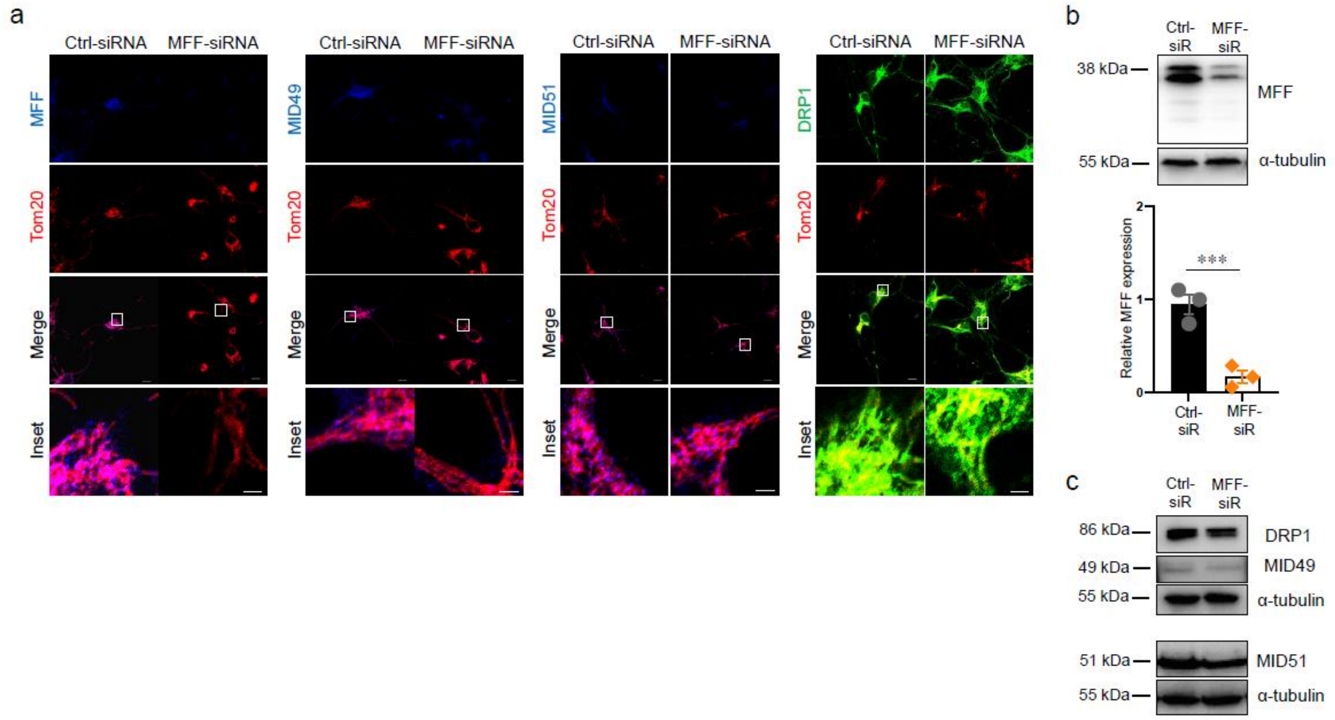

Figure S1. (**a**) DNs were stained with mouse monoclonal anti-Tom20 (#sc-17764; Santa Cruz Biotechnology, Paso Robles, CA, USA), rabbit polyclonal anti-MFF (#170909-1-AP; Proteintech, Rosemont, IL, USA), rabbit polyclonal anti-MID51 (#20164-1-AP; Proteintech), rabbit polyclonal anti-MID49 (#16413-1-AP; Proteintech), and mouse monoclonal anti-DRP1 (#611113; BD Biosciences, San Jose, CA, USA) antibodies. Scale bars = 10 μm. The boxed regions on the merged images are shown at a greater magnification in the lower panels. Scale bars = 2 μm. (**b**) MFF protein levels were measured by western blotting using anti-MFF and mouse monoclonal anti-α-tubulin (#sc-32293; Santa Cruz Biotechnology) antibodies. The mean ± SEM was taken from three independent experiments. ***$p < 0.001$. (**c**) DRP1, MID49, and MID51 protein levels were detected by western blotting using the aforementioned antibodies.

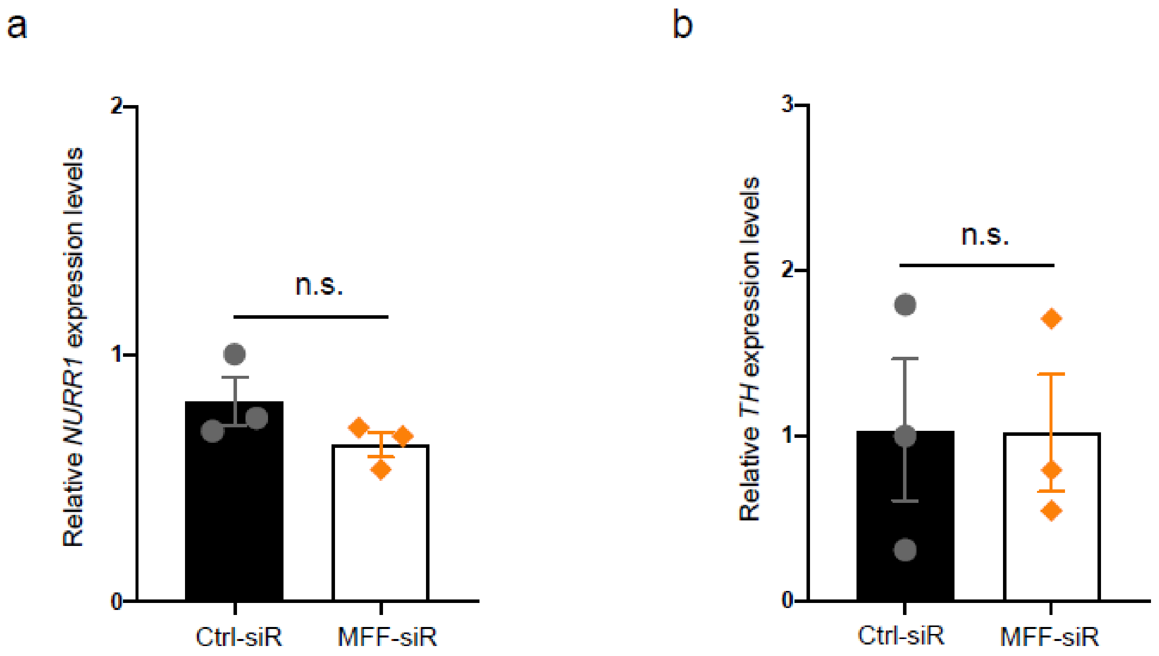

Figure S2. *NURR1* and *TH* mRNA expression in DNs were measured using RT-qPCR. (**a**) The primer set for *NURR1* is forward 5'-GCACTTCGGCAGAGTTGAATGA-3' and reverse 5'-GGTGGCTGTGTTGCTGGTAGTT-3'. (**b**) The primer set of *TH* was purchased from Bio-Rad (#qHsaCED0001111; Bio-Rad, Hercules, CA, USA). Relative expression of the target gene was analyzed using the comparative threshold cycle method by normalizing to 18s expression. The mean ± SEM was taken from three independent experiments. n.s., not significant.

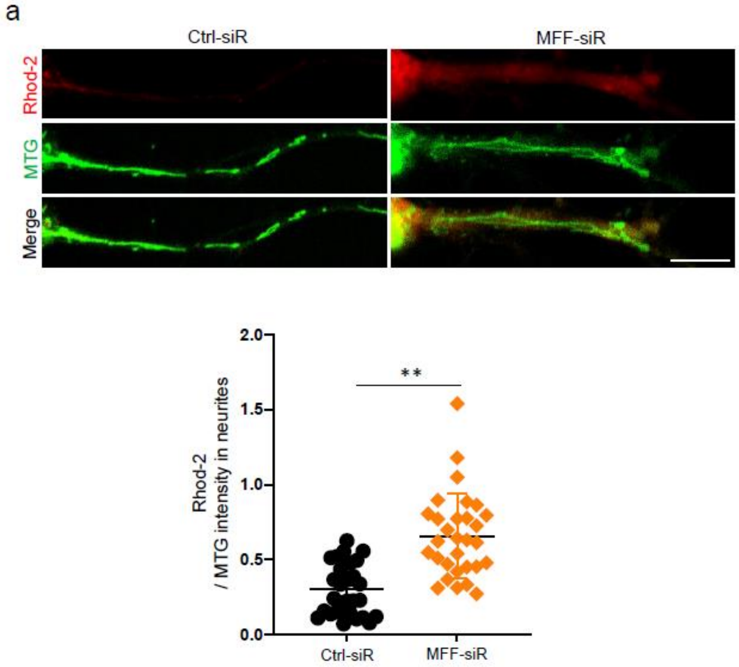

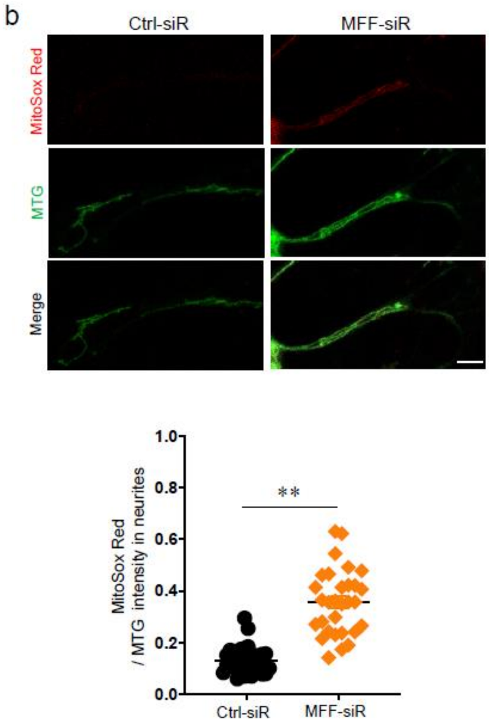

Figure S3. (**a**) DNs were stained with Rhod-2 AM and MTG. Mitochondria in neurites were observed using confocal microscopy. Scale bar = 10 μm. To measure the mitochondrial Ca^{2+} level per mitochondrion in neurites, fluorescence intensity of 10 mitochondria in each case were measured and Rhod-2 AM intensity was divided by that of MTG. The mean ± SEM was taken from three independent experiments. $^{**}P < 0.01$. (**b**) DNs were stained with MitoSOX Red and MTG. Mitochondria in neurites were observed using confocal microscopy. Scale bar = 5 μm. To measure the ROS level per mitochondrion in neurites, the fluorescence intensities of 10 mitochondria in each case were measured and MitoSOX Red intensity was divided by that of MTG. The mean ± SEM was taken from three independent experiments. $^{**}P < 0.01$.

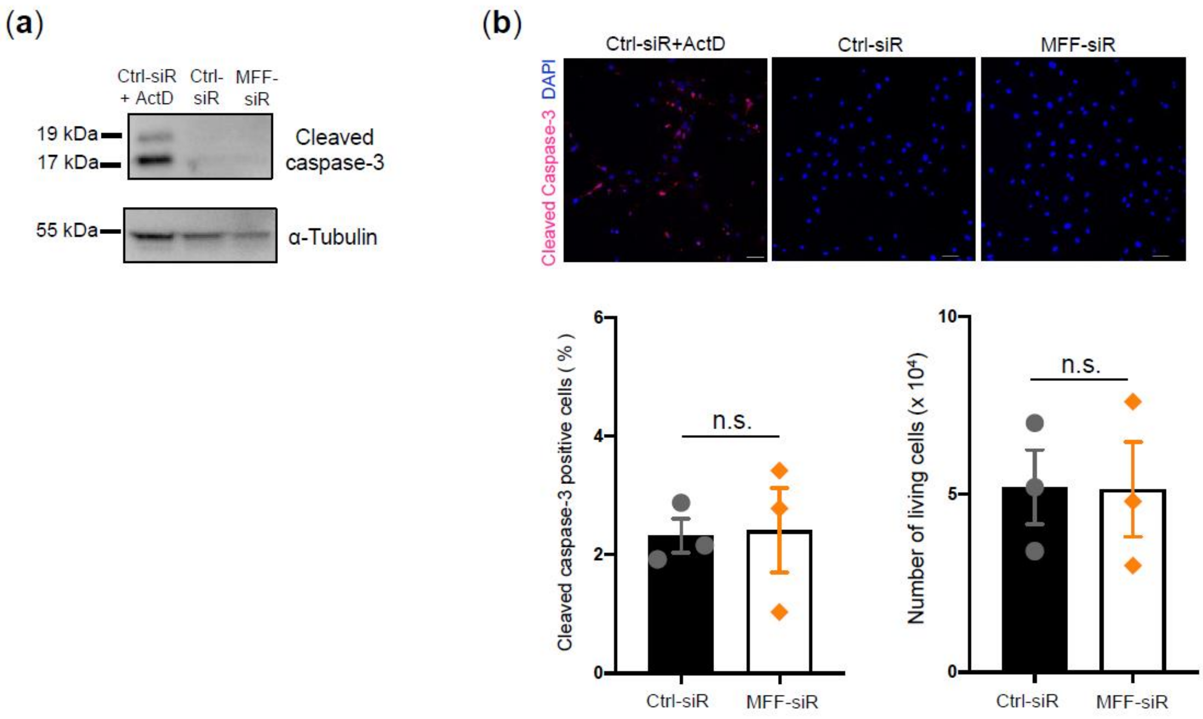

Figure S4. (**a**) Cleaved caspase-3 expression in DNs was detected using western blotting. The protein sample of control cells treated with 5 μM actinomycin D (ActD) for 12 h was used as a positive control for apoptotic cells. (**b**) DNs were stained with mouse monoclonal anti-cleaved caspase-3 antibody (#9664S; Cell Signaling Technology, Danvers, MA, USA) and DAPI. Scale bars = 50 μm. The left graph shows the percentage of cleaved caspase-3-positive cells; 100 cells were counted for each case. DNs were stained with trypan blue solution to distinguish between live and dead cells. Graph on the right shows the number of living cells. The mean ± SEM was taken from three independent experiments. n.s., not significant.

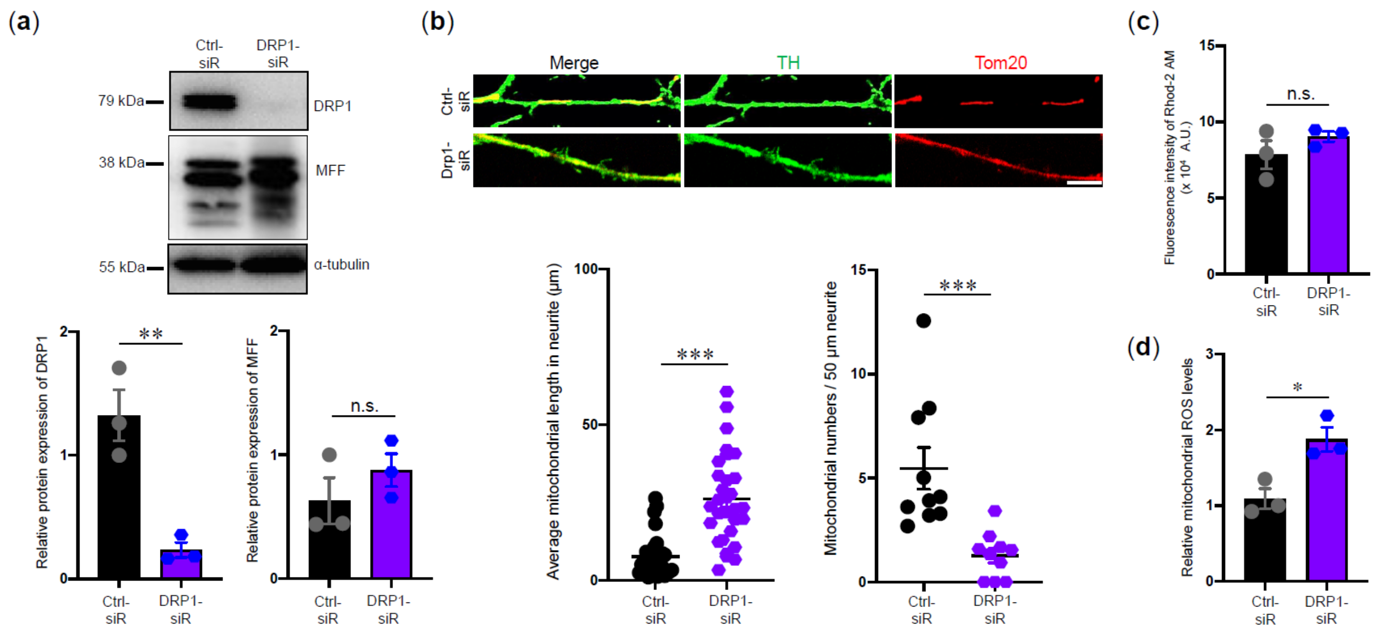

Figure S5. (a) SHEDs were transfected with negative control- (Ctrl-siR) and DRP1-siRNA (DRP1-siR) and differentiated into DNs. The DRP1 siRNA sequences were as follows: sense 5'-GUAAUACUGAGACUUUGUUdTdT-3' and antisense 5'-AACAAAGUCUCAGUAUUACdTdT-3'. The control siRNA was purchased from Sigma-Aldrich, St. Louis, MO, USA (SIC001-10NMOL). DRP1 and MFF levels were measured using western blotting with anti-MFF (#170909-1-AP; Proteintech), mouse monoclonal anti-DRP1 (#611113; BD Biosciences), and mouse monoclonal anti-α-tubulin (#sc-32293; Santa Cruz Biotechnology) antibodies. The mean ± SEM was from three independent experiments. n.s., not significant, **$p < 0.001$. (b) DNs were stained with anti-Tom20 and anti-TH antibodies and counterstained with DAPI. Mitochondrial proportion in neurites was observed using confocal microscopy. Scale bar = 5 μm. Mitochondrial length in neurites was measured for 10 mitochondria of each case (left graph), and the number of mitochondria per 50 μm neurite was measured for 10 neurites of each case (right graph). The mean ± SEM was taken from three independent experiments. ***$p < 0.001$. (c) DNs were stained with Rhod2-AM. Fluorescence intensity was measured using a plate reader. The mean ± SEM was taken from three independent experiments. n.s., not significant. (d) DNs were stained with MitoSOX Red, and the signal was measured using flow cytometry. The mean ± SEM was taken from three independent experiments. *$p < 0.05$.

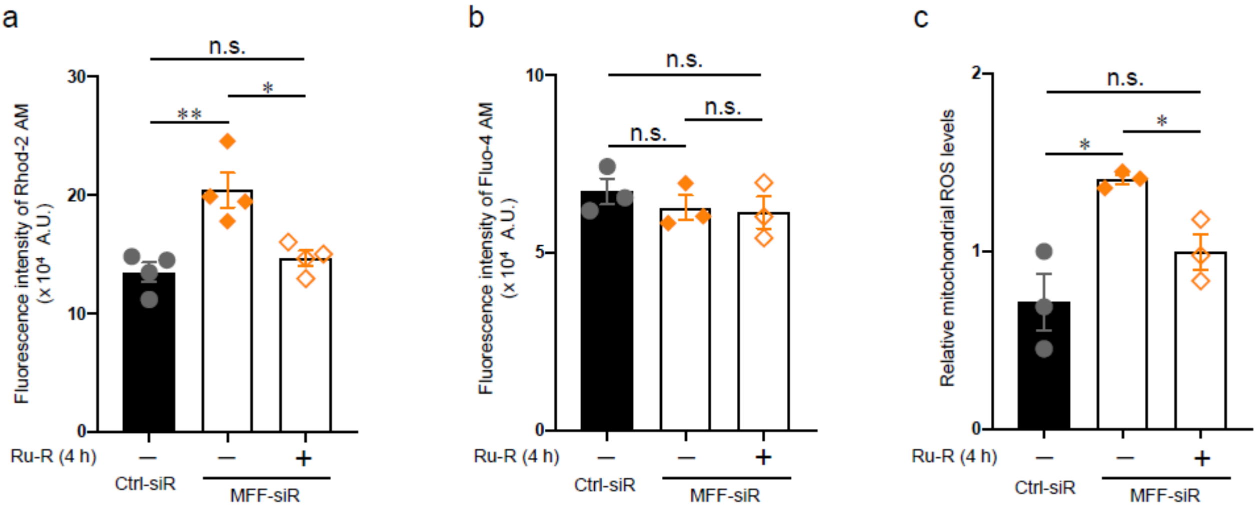

Figure S6. DNs were treated with 1 μM Ru-R for the last 4 h of culture. (**a**, **b**) DNs were stained with Rhod-2 AM or Fluo-4 AM. Fluorescence intensities of these fluorescent probes were measured using a plate reader. The mean ± SEM was taken from three independent experiments. n.s., not significant, *p < 0.05, **p < 0.01. (**c**) DNs were stained with MitoSOX Red, and the signal was measured using flow cytometry. The mean ± SEM was taken from three independent experiments. n.s., not significant, *p < 0.05.

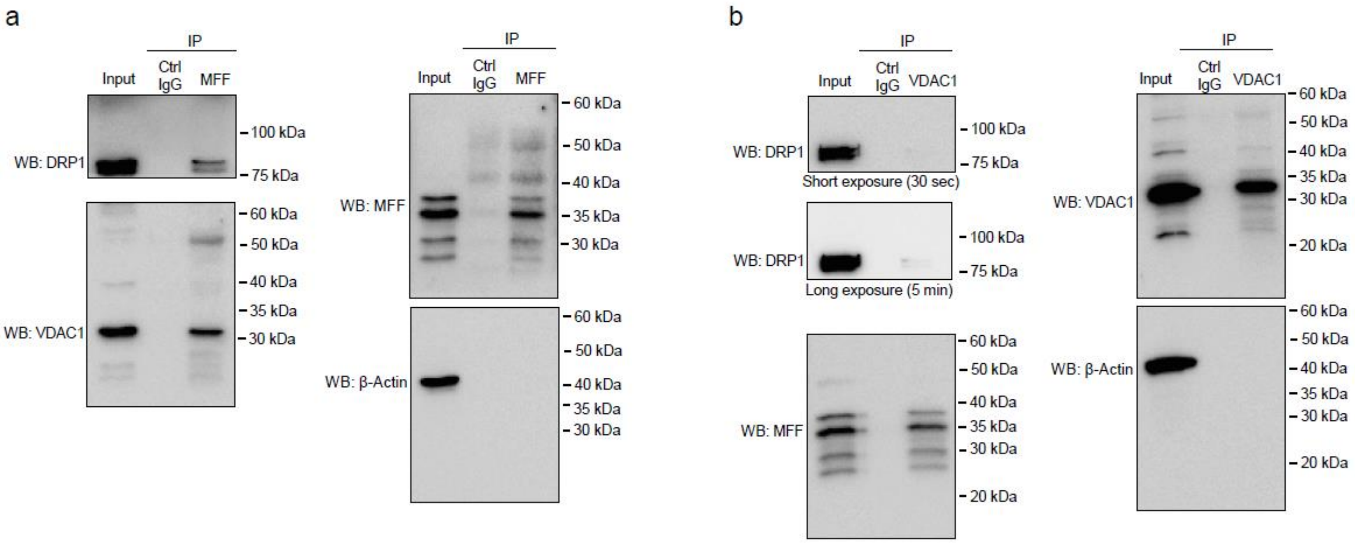

Figure S7. Full scans of the western blots from Figure 4c. (**a**, **b**) IP was performed using an anti-MFF and anti-VDAC1 antibodies. Immunoprecipitants were detected by western blotting (WB) using the indicated antibodies.

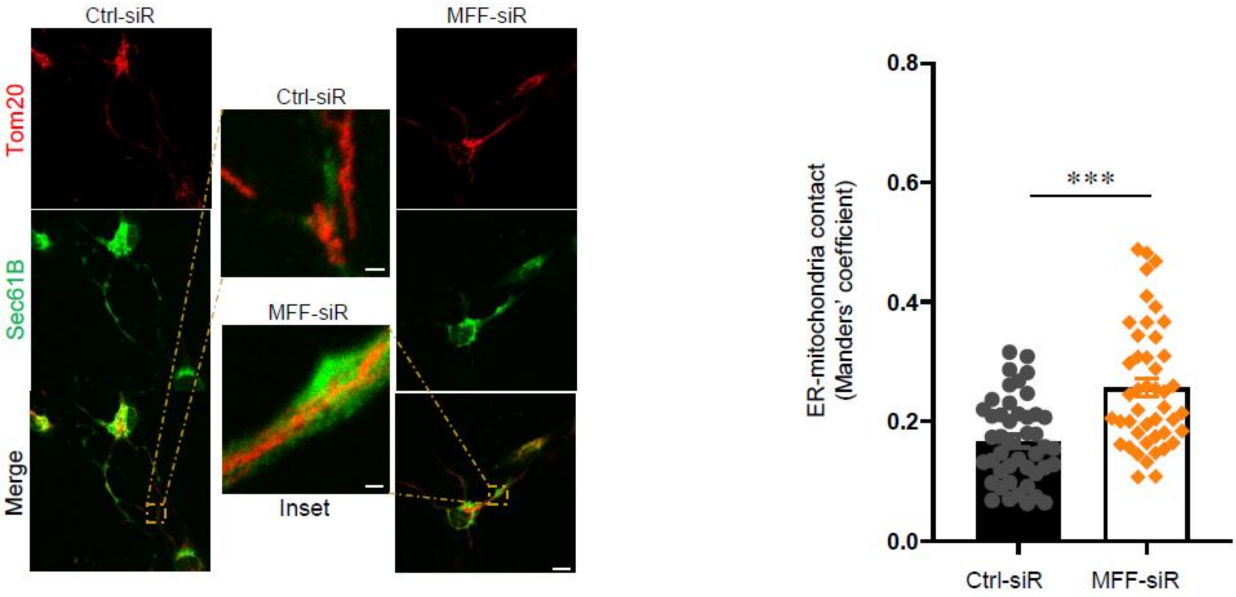

Figure S8. Immunofluorescence analysis of mitochondria and ER contact. DNs were stained with mouse monoclonal anti-Tom20 (#sc-17764; Santa Cruz Biotechnology) and rabbit polyclonal anti-Sec61B (#14648S-AP; Cell Signalling Technology) antibodies. Scale bar = 20 μm. The boxed regions on the merged images are shown at a greater magnification in the lower panels. Scale bars = 2 μm. ER and mitochondrial colocalization were analyzed through Mander's co-localization coefficients using ImageJ software version 1.53 with JACop plugin. Fifteen DNs were analyzed in each case. The mean ± SEM was taken from three independent experiments. ***$p < 0.001$.

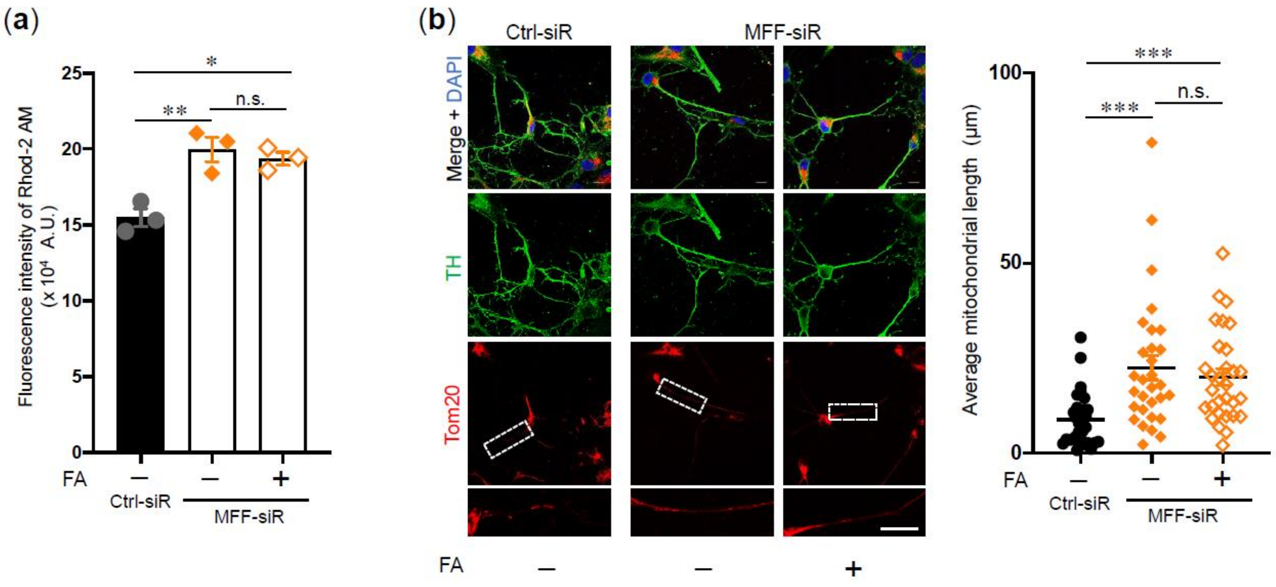

Figure S9. (**a**) DNs were stained with Rhod-2 AM. The fluorescence intensity of the probe was measured using a plate reader. The mean ± SEM was taken from three independent experiments. n.s., not significant, *p < 0.05, **P < 0.01. (**b**) DNs were stained with anti-Tom20 and anti-TH antibodies and counterstained with DAPI. Scale bars = 10 μm. The boxed regions on the Tom20-stained images are shown at a greater magnification in the lower panels. Scale bar = 5 μm. Average mitochondrial length in neurite was measured for 10 mitochondria of each case. The mean ± SEM was taken from three independent experiments. n.s., not significant, ***p < 0.001.
